# La maladie cœliaque: une cause rare de fausses couches à répétition

**DOI:** 10.11604/pamj.2016.25.197.10096

**Published:** 2016-11-28

**Authors:** Mehdi Kehila, Rim Ben Hmid, Imene Godcha, Hassine Saber Abouda, Oueslati Boujomaa, Mohamed Badis Chanoufi

**Affiliations:** 1 Service C de Gynécologie Obstétrique, Centre de Maternité et de Néonatologie de Tunis, Université Tunis El Manar, Tunisie

**Keywords:** Maladie coeliaque, grossesse, fausse couche, maladie abortive, Cœliac disease, pregnancy, miscarriage, abortive illness

## Abstract

La maladie cœliaque est un trouble auto-immun associé à une intolérance au Gluten qui a pour effet la destruction progressive des villosités de l’intestin grêle. Les symptômes de la maladie cœliaque sont très divers et peuvent se produire à tout âge. Parmi ces symptômes, la maladie abortive est une circonstance rare de découverte de la maladie cœliaque. Nous rapportons le cas d’une patiente ayant présenté 12 fausses couches successives dont l’étiologie a été enfin rattachée à une maladie cœliaque.

## Abstract (en)

Cœliac disease is an autoimmune disorder associated with Gluten intolerance resulting in progressive destruction of the villi of the small intestine. Symptoms are very diverse and can occur at any age. Abortive illness is a rare symptom leading to the detection of cœliac disease. We report the case of a patient with a history of 12 consecutive miscarriages whose etiology was finally related to cœliac disease.

## Introduction

La maladie cœliaque est un trouble auto-immun dépendant de facteurs génétiques qui a pour effet la destruction progressive des villosités de l’intestin grêle par certains peptides du blé, du seigle et de l’orge (communément appelés le gluten). Les symptômes de la maladie cœliaque sont très divers et peuvent se produire à tout âge [1]. Les fausse couches à répétition, bien que décrites dans la littérature comme circonstance de découverte de la maladie cœliaque [2], sont un symptôme devant lequel on n’évoque pas forcément à cette pathologie. Nous rapportons le cas d’une patiente ayant présenté 12 fausses couches successives largement explorée avant de les relier enfin à une maladie cœliaque.

## Patient et observation

Il s’agit d’une patiente âgée de 41 ans groupe sanguin O positif ayant consulté pour un problème de fausses couches précoces à répétition. Dans les antécédents de la patiente on note : Une allergie à la pénicilline, 2 fractures osseuses récentes une au poignet et une à la cheville. Les 2 survenant à la suite d’une simple chute de sa propre hauteur. La patiente a déjà mené une première grossesse à terme il y a 9 ans, donnant naissance à un petit garçon en bonne santé. La patiente a eu par la suite une succession de 12 grossesses arrêtées au premier trimestre documentées. Dans le cadre du bilan étiologique de cette maladie abortive, ces explorations ont été réalisées: un dépistage de diabète; une recherche de thrombophilie (Anticoagulants circulant, déficit en anti thrombine, anticorps anti DNA natif, anticorps anti nucléaires, déficit en protéine C, déficit en protéine S, recherche de syndrome des anticorps antiphospholipides, recherche d’une hyperhomocystéinémie); un caryotype parental; une hystérosalpingographie ; les examens anatomopathologiques des produits d’avortements; un spermogramme; une recherche d’agglutinines irrégulières; un bilan infectieux: prélèvement vaginal.

Tous ces bilans étaient normaux. A noter que 3 parmi ces grossesses arrêtées ont été menées sous Aspégic 100 à la dose d’un sachet par jour et les deux dernières sous une association d’Aspégic-HBPM. Nous avons poussé par la suite l’interrogatoire concernant les 2 fractures récentes, cherchant des éléments en faveur d’une ostéoporose. C’est alors que la patiente nous explique que ses fractures sont probablement expliquées par une maladie cœliaque qu’elle a omis de nous révéler. Cette maladie a été diagnostiquée à l’âge de 26 ans suite à des diarrhées récidivantes. La patiente nous révèle qu’après son accouchement elle a commencé à faire quelques écarts du régime sans gluten vu que, d’une part, les aliments dépourvus de gluten étaient chers pour elle, et que d’autre part elle se sentait bien à part quelques épisodes de diarrhées. Elle a fini de ne plus suivre ce régime. A l’examen physique, la patiente présentait seulement quelques signes mineurs de carence vitaminique notamment une chute de cheveux et des ongles cassants. Son indice de masse corporel était normal. Nous avons expliqué à la patiente que la maladie cœliaque pouvait être une étiologie de fausses couches à répétition et qu’il était primordial de bien suivre le régime sans Gluten. La patiente a depuis commencé à suivre le régime sans Gluten. Trois mois plus tard, la patiente a eu une nouvelle grossesse, elle a poursuivi son régime tout au long de la grossesse et elle a été aussi mise sous l’association Aspégic-HBPM. La grossesse s’est déroulée sans incidents et elle a accouché d’une fille en bon état de santé de 3150g.

## Discussion

En 1970, Moriss et al. étaient les premiers a rapporté le cas de trois femmes infertiles chez qui la découverte ainsi que le traitement de la maladie cœliaque ont permis d’obtenir des grossesses et des enfants en bon état de sante [3]. Bien que décrits, depuis, dans la littérature comme symptômes fréquents de la maladie cœliaque, les troubles de la vie génitale de la femme font rarement chercher cette pathologie [4, 5]. En effet, 19.4% des femmes atteintes de cette maladie ont des aménorrhées mais aussi, des oligo-hypo-ménorrhées, dysménorrhées et des métrorragies [6]. Il existe aussi une forte corrélation entre maladie cœliaque et fausses couches précoces, menaces d’avortement, toxémie gravidique et retard de croissance intra-utérin [7]. La pathogénie de tous ces problèmes gynéco-obstétricaux reste mal connue, mais les hypothèses sont partagées entre l’origine auto-immune et la malnutrition [8]. Dans le cadre de la recherche de la physiopathologie de ces troubles, certains auteurs se sont intéressés à des vitamines et oligoéléments comme l’acide folique, le sélénium et le zinc. Ceux-ci sont des nutriments essentiels pour la fonction reproductive de la femme, et sont malabsobés en cas de maladie cœliaque [2]. Ces auteurs ont montré, entre autres, que le déficit en zinc engendre un trouble dans la sécrétion et l’action de la LH et celle de la FSH ce qui perturbe le fonctionnement ovarien. Ceci peut expliquer en partie les problèmes d’infertilité et de fausses couches précoces engendrés par la maladie cœliaque. Se basant sur cette hypothèse physiopathogénique, certains auteurs sont parvenus à rétablir une bonne fonction reproductive en corrigeant ces déficits parallèlement à un régime sans gluten chez des femmes infertiles atteintes de la maladie cœliaque [9]. Toutefois, d’autres études ont montré que la simple correction de ce déficit nutritif, sans associer un régime sans Gluten, ne suffisait pas pour obtenir des grossesses. Les auteurs ont conclu que le problème d’infertilité associé à la maladie cœliaque est plus complexe qu’un simple déficit en vitamines et oligo-éléments [10, 11]. L’autre piste physiopathologique explorée pour essayer de comprendre le mécanisme de ces perturbations de la vie génitale associées à la maladie cœliaque est celle de l’auto-immunité. Dans ce sens, certains auteurs ont comparé des sérums de femmes en bonne santé à ceux de femmes atteintes de maladie cœliaque active avec des manifestations gynéco-obstétricales. Ils ont prouvé qu’il existait une relation entre la présence d’anticorps anti endomysium et anti transglutaminase et ces manifestations cliniques [12]. Ce cas clinique vécu, illustre bien l’association entre la maladie cœliaque et les fausses couches à répétition. Il montre bien aussi la difficulté qu’a le clinicien à rattacher le symptôme « maladie abortive » à cette pathologie. Enfin, le cas que nous présentons conforte l’avis de nombreux auteurs qui considèrent que la maladie cœliaque non traitée est plus pourvoyeuse de ces complications [13]. En effet, il a été démontré une corrélation entre le taux plus élevé d’anticorps en cas de maladie cœliaque active et les manifestations cliniques gyneco-obstetricales [14–16].

## Conclusion

Ce cas illustre bien la relation entre maladie cœliaque et maladie abortive. Il doit inciter le clinicien à chercher, en cas de fausses couches à répétition, des signes digestifs mineurs ou des signes de malabsorption pouvant évoquer la maladie cœliaque. La physiopathologie de cette maladie est mal connue mais ce qui est actuellement admis est l’existence d’une part d’une composante de malabsorption mais surtout d’une composante d’auto-immunité. Le diagnostic de cette maladie en cas de manifestations gynéco-obstétricales est primordial surtout qu’un simple régime sans Gluten permet souvent de résoudre le problème.
